# Structural equation modeling of multidimensional determinants of postoperative quality of life in patients with oral cancer

**DOI:** 10.1186/s12955-026-02476-1

**Published:** 2026-01-22

**Authors:** Ziyan Xiong, Jiajing Zhou, Xiaohui Wang, Jiayao Huang, Chunni Lin, Li Cong

**Affiliations:** 1 Hunan Normal University Health Science Center, 371 Tongzipo Road, Changsha, 410013 China; 2 Kiang Wu Nursing College of Macau, Macao, Macao SAR 999078 China

**Keywords:** Quality of life, Oral cancer, Oncology, Postoperative care, Structural equation modeling

## Abstract

**Background:**

Postoperative quality of life (QoL) in oral cancer patients is not only influenced by physical impairments but also by interrelated psychosocial factors. However, few studies have investigated these multidimensional pathways during the acute recovery phase. This study aimed to examine how speech disorder, self-esteem, social alienation, and body image interact in relation to QoL in the first week following oral cancer surgery.

**Methods:**

A cross-sectional study was conducted among 401 patients within 7 days post-surgery. Validated Chinese versions of standardized scales were administered to assess functional and psychosocial domains relevant to QoL. Structural equation modeling was used to test hypothesized pathways among speech disorder, self-esteem, social alienation, body image, and quality of life. Model fit was assessed with χ²/df, CFI, TLI, RMSEA, and SRMR, using established thresholds.

**Results:**

Speech disorder was negatively associated with QoL both directly (β = −0.446) and indirectly through sequential pathways involving self-esteem, social alienation, and body image. Among these variables, speech disorder showed the strongest overall association with QoL when both direct and indirect pathways were considered (β = −0.824), whereas body image demonstrated the strongest direct association with QoL (β = −0.609). The structural equation modeling demonstrated acceptable model fit (χ² = 187.818, df = 78, χ²/df = 2.408, CFI = 0.969, TLI = 0.959, RMSEA = 0.059, SRMR = 0.039).

**Conclusions:**

Postoperative QoL in oral cancer patients is shaped by complex mediation pathways linking speech dysfunction, psychosocial vulnerabilities, and body image. These findings highlight the need for early multidisciplinary interventions that integrate functional rehabilitation with psychosocial support to improve recovery. Future research is warranted to validate these associations through longitudinal and interventional studies.

**Supplementary Information:**

The online version contains supplementary material available at 10.1186/s12955-026-02476-1.

## Introduction

Oral cancer represents a significant global health burden, ranking as the 16th most prevalent malignancy worldwide, with over 350,000 new cases and 150,000 deaths reported annually [1]. The etiology of oral cancer is multifactorial, with established risk factors including tobacco use (both smoking and smokeless forms) [2], alcohol consumption, betel quid chewing, excessive sun exposure, passive smoking, and infection with human papillomavirus (HPV) [3, 4]. In China, while smoking and alcohol use are major risk factors for oral cancer, betel quid chewing is particularly significant [5, 6]. This habit is most prevalent in Hunan, Hainan, and Yunnan provinces, with Hunan showing the highest rates [7]. Surgical resection remains the cornerstone of treatment for oral cancer patients [8]. However, both the disease and its treatments often result in lasting impairments in physical appearance, speech function, and psychological well-being [9–11], leading to a substantial decline in quality of life (QoL).

While advances in detection and therapy have improved survival rates, extended survival is no longer the sole endpoint of cancer care. Increasingly, attention is turning to how well patients live after treatment, not just how long [12, 13].Yet, current evidence remains limited, as many studies have prioritized clinical indicators, and investigations of psychosocial and functional dimensions, although increasing, are still insufficient to capture the complexity of survivorship experiences. Emerging literature suggests that constructs such as “body image”, “speech function”, “self-esteem”, and “social alienation” significantly influence QoL in head and neck cancer survivors [14, 15]. In this study, the construct of body image refers to negative perceptions and distress related to appearance and function. These variables not only reflect physical status but also encapsulate the emotional and social challenges patients face in daily life. Taken together, these observations underscore the need to better understand how functional and psychosocial domains jointly shape survivorship in oral cancer.

Several studies have examined associations among speech function, body image, self-esteem, and QoL in head and neck cancer populations. For example, speech and swallowing problems have been associated with poorer body image and reduced QoL [16, 17]. Body image concerns have also been linked to psychological distress and self-esteem, highlighting the interplay between psychosocial and functional domains [18, 19]. Evidence further suggests that self-esteem contributes to postoperative QoL outcomes, with lower self-esteem predicting poorer recovery [20]. More recently, structural equation modeling (SEM) has been used to examine pathways among functional and psychosocial factors, providing empirical support for integrated frameworks [21].

Although immediate postoperative declines in QoL have been reported after head and neck cancer surgery [22], evidence specifically addressing the first postoperative week remains scarce [23]. Notably, studies have documented that within 7 days after surgery, patients experience poorly controlled pain, heavy symptom burden, and markedly reduced physical functioning [24, 25]. This acute phase may strongly affect body image and exert lasting influence on rehabilitation outcomes [26, 27]. Accordingly, this study aims to generate context-specific evidence by applying an SEM framework to delineate the integrated pathways linking speech disorder, body image, self-esteem, social alienation, and QoL in the first week after surgery. Understanding these early dynamics may inform timely interventions to improve long-term rehabilitation outcomes.

## Methods

### Design

This study employed a cross-sectional, descriptive design to examine QoL and their associated factors among postoperative oral cancer patients. A quantitative approach was adopted, with structural equation modeling applied to examine both direct and indirect pathways among the measured psychosocial and functional variables.

### Setting and sample

Participants were recruited through convenience sampling from the Department of Head and Neck Surgery at a tertiary hospital in Changsha, China, between October 2023 and August 2024. The inclusion criteria required: (1) histologically confirmed primary oral carcinoma [28], with different subsites analyzed together because they are clinically considered part of the same oral cavity cancer entity and show broadly comparable postoperative functional and psychosocial patterns [29–31]. (2) status post-surgical resection within 7 days, and (3) preserved cognitive and communicative capacity. Exclusion criteria eliminated patients with pre-existing deformities, recurrent malignancies, or severe psychiatric comorbidities. A priori sample size calculation determined that 297–594 participants were needed to achieve adequate power for SEM analysis (10–15 cases per observed variable) [32, 33], accounting for a 10% attrition rate.

### Data collection tools

#### General information questionnaire

Designed by the research team based on a literature review and the study objectives, this questionnaire consists of 17 items covering two sections: sociodemographic information and disease-related data. The sociodemographic section includes variables such as gender, age and type of health insurance (in the Chinese context, including urban employee insurance, urban and rural resident insurance, commercial insurance, or self-payment). While the disease-related section includes factors such as lesion location, pathological type, and disease stage.

#### Body image scale (BIS)

BIS, developed by Hopwood et al. in 2001 [34], has been widely adopted for assessing body image disturbances in cancer populations. This instrument comprises 10 items measuring three distinct dimensions. Higher scores indicate more severe body image disturbances, with a cutoff score of ≥ 10 indicating clinically significant body image disturbance. The original version demonstrated excellent reliability and validity, with Cronbach’s Alpha of 0.93. The validated Chinese version has also shown good psychometric properties in cancer patients [35]. Cronbach’s Alpha was 0.844 in our study. In this manuscript, the term “body image” is used to denote body image disturbance as operationalized by the BIS.

#### Speech handicap index (SHI)

SHI, was developed by Rinkel et al. in 2008 [36] and comprises two dimensions: psychosocial impact and speech function. The scale consists of 30 items, each rated on a 5-point Likert scale ranging from 0 to 4, with a total score ranging from 0 to 120. The Chinese version of the scale was translated and validated by Wu Peixia et al. in 2014 [37], with Cronbach’s Alpha of 0.91, and good validity. In the present study, Cronbach’s Alpha for this scale was 0.968.

#### General alienation scale (GAS)

GAS, developed by Jessor et al. in 1973 [38], this scale is primarily used to assess an individual’s sense of isolation and uncertainty in social participation. It comprises four dimensions: alienation from others, distrust, self-alienation, and a sense of meaninglessness, with a total of 15 items. The total score ranges from 15 to 60, with higher scores indicating a greater degree of social alienation. The Chinese version revised by Wu Shuang et al. demonstrated acceptable reliability and validity in clinical samples, with Cronbach’s Alpha of 0.77 [39]. Cronbach’s Alpha was 0.871 in our study.

#### Self-esteem scale (SES)

SES, was developed by Rosenberg in 1965 [40] and later adapted into a Chinese version by Ji Yifu et al. [41]. It has been widely used in China to assess self-esteem across different populations. The scale consists of 10 items rated on a 4-point Likert scale, with a total score ranging from 10 to 40. Higher scores indicate a higher level of self-esteem. Cronbach’s Alpha for this scale was 0.88, the test-retest reliability was 0.82, and Cronbach’s Alpha was 0.852 in this study.

#### University of Washington quality of life questionnaire (UW-QOL)

UW-QOL, was a self-administered questionnaire specifically designed to assess the health-related QoL in patients with head and neck cancer. Since its initial release in 1993, it has undergone multiple revisions, with the latest version being the fourth edition [42].The UW-QOL has demonstrated good reliability and validity internationally, with Cronbach’s Alpha ranging from 0.81 to 0.85 in the total sample. The validated Chinese version also showed satisfactory psychometric properties [43]. Cronbach’s Alpha was 0.816 in this study.

### Data analysis

This study utilized Epidata 3.1 for data entry, with dual verification to ensure data accuracy. Then utilized IBM SPSS Statistics 29.0 and IBM SPSS Amos 28.0 for statistical analysis. (1) The reliability of the questionnaire used in the study was assessed using Cronbach’s Alpha to examine internal consistency. The Kaiser-Meyer-Olkin (KMO) test and Bartlett’s test of sphericity were used to assess sampling adequacy and the suitability of the data for factor analysis. Construct validity was examined through exploratory factor analysis (EFA), with evidence derived from the cumulative variance explained and the factor loading patterns of each scale. (2) QoL scores were used as the dependent variable, while 15 items from the general information questionnaire were treated as independent variables. Due to non-normal distribution of QoL scores, nonparametric tests were used to assess group differences. Mann-Whitney U tests were applied for comparisons between two groups, and Kruskal-Wallis H tests were used for comparisons involving three or more groups. (3) A multivariable linear regression model including all 15 demographic and clinical variables was conducted to examine their associations with QoL and to provide empirical information on variables that could be considered as potential covariates in subsequent modeling. (4) Pearson correlation analysis was performed to explore the relationships between body image, social isolation, speech impairment, self-esteem, and QoL. (5) Although the SES and UW-QOL are validated instruments with established structures, confirmatory analyses in this early postoperative oral cancer sample showed suboptimal fit. To ensure the latent constructs in the SEM reflected the observed data, EFA was conducted using principal component analysis with varimax rotation. Factor retention was based on a combination of criteria, including eigenvalues close to or greater than 1, scree plot inspection, cumulative variance explained, and theoretical interpretability. All items were retained, and the resulting dimensions were incorporated as latent constructs in the SEM. (6) SEM was constructed using IBM SPSS Amos 28.0 to examine the pathway relationships and effects of QoL in postoperative oral cancer patients. Given our a priori focus on psychosocial pathways and the markedly unbalanced distribution of certain subgroups, only variables identified as clinically meaningful in subsequent analyses were considered as potential covariates. The SEM therefore tested hypothesized links among speech disorder, body image, self-esteem, social alienation, and QoL. Model fit was evaluated with multiple indices using commonly recommended thresholds: the Goodness-of-Fit Index (GFI), Adjusted Goodness-of-Fit Index (AGFI), Normed Fit Index (NFI), Comparative Fit Index (CFI), Incremental Fit Index (IFI), Tucker-Lewis Index (TLI), Root Mean Square Error of Approximation (RMSEA), and Standardized Root Mean Square Residual (SRMR). Values of χ²/df < 3, GFI/AGFI/NFI/CFI/IFI/TLI > 0.90, and RMSEA/SRMR < 0.08 were considered acceptable [44]. (7) Indirect effects were evaluated within the SEM via the product-of-coefficients approach and summarized with standardized indirect and total effects.

## Results

### Descriptive statistics

**Fig. 1:**
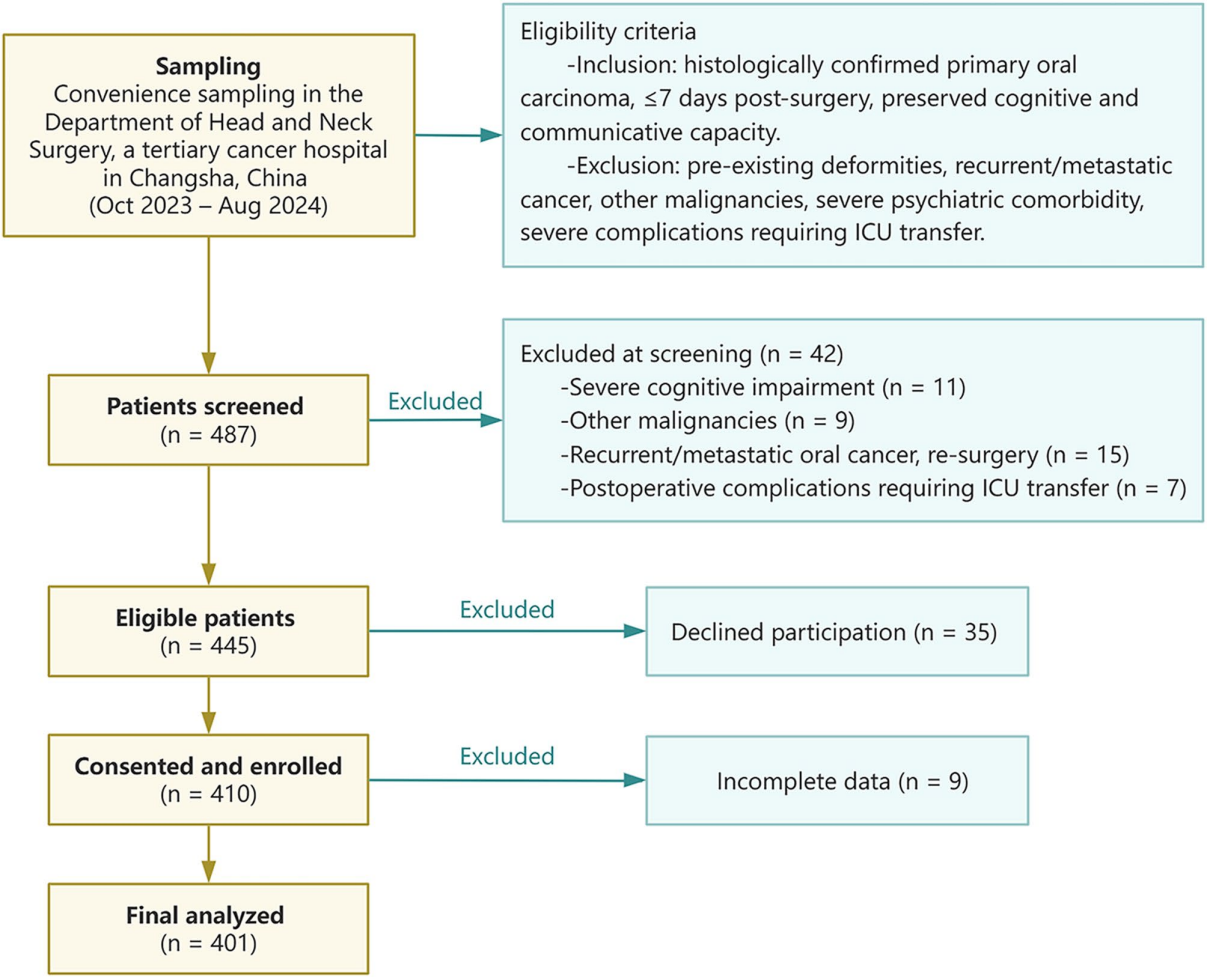
Flowchart of patient recruitment and enrollment

A total of 487 patients were screened, of whom 445 were eligible. Among these, 410 consented and 401 completed the study (Fig. 1), yielding an enrollment rate of 91.4% among eligible patients, an overall rate of 82.3%, and a retention rate of 97.8%. Table 1 presents the distribution of demographic and clinical characteristics, along with the corresponding QoL scores expressed as means and standard deviations (M ± SD). The sample was predominantly male (90.8%) and married (94.8%), with the most common age group being 51–60 years (43.4%). A majority resided in rural areas (53.1%), and 46.1% had completed junior high school. More than half of the participants were unemployed (58.1%), and 55.6% identified their spouse as their primary caregiver. Clinically, 96.3% of the participants were diagnosed with squamous cell carcinoma, with the tongue (54.1%) being the most common lesion site. Most patients were classified at Stage II (34.7%), and 72.1% had not received adjuvant therapy. Additionally, 72.8% had no reported comorbidities. As the distribution of QoL scores and related variables did not meet the assumptions of normality, nonparametric methods were applied. Significant differences in QoL were found by Mann-Whitney U and Kruskal-Wallis H tests for marital status, residential method, caregiver identity, tumor stage, chemoradiotherapy, and complications (all *P* < 0.05). Tumor stage had the greatest impact, with Stage IV patients reporting the lowest QoL (M = 58.21, SD = 14.02).

**Table 1:** Demographic characteristics related to quality of life in postoperative oral cancer patients (*N* = 401)

Variables	*N*	Proportion (%)	Quality of life (M ± SD)	U / H	*P*
Age				*H* = 2.079	0.556
30 ~ 40	47	11.7	66.7 ± 10.9		
41 ~ 50	116	28.9	65.12 ± 13.08		
51 ~ 60	174	43.4	66.99 ± 10.36		
>60	64	16	64.06 ± 12.53		
Gender				*U* = 5435.000	0.053
Male	364	90.8	66.38 ± 10.97		
Female	37	9.2	61.68 ± 16.46		
Marital status				*U* = 2922.000	0.039
Married	380	94.8	66.22 ± 11.62		
Unmarried/ Divorced/ Widowed	21	5.2	61.11 ± 11.03		
Habitation				*U* = 20882.000	0.458
City	188	46.9	65.66 ± 11.99		
Countryside	213	53.1	66.21 ± 11.34		
Residential method				*U* = 3069.000	0.008
Live with spouse/Children	377	94	66.29 ± 11.66		
Solitude	24	6	60.55 ± 10.03		
Personal monthly income (RMB)				*H* = 4.754	0.191
<1000	85	21.2	64.04 ± 11.79		
1000 ~ 2999	127	31.7	66.12 ± 11.79		
3000 ~ 4999	99	24.7	66.10 ± 11.15		
≥ 5000	90	22.4	67.36 ± 11.76		
Educational level				*H* = 0.784	0.853
Primary and below	69	17.2	65.55 ± 10.54		
Junior high school	185	46.1	65.65 ± 11.69		
High school / Technical secondary	99	24.7	66.66 ± 11.53		
College or above	48	12	66.20 ± 13.32		
Occupation				*H* = 2.032	0.362
Employed	129	32.2	66.90 ± 11.64		
Unemployed	233	58.1	65.61 ± 11.42		
Retire	39	9.7	64.80 ± 12.95		
Caregiver identity				*H* = 15.01	0.002
Self	134	33.4	67.01 ± 10.95		
Spouse	223	55.6	66.36 ± 11.56		
Child	31	7.7	57.91 ± 12.44		
Other	13	3.2	67.15 ± 11.85		
Type of health insurance				*H* = 4.555	0.207
Self-payment	17	4.2	63.77 ± 12.18		
Urban employee insurance	98	24.4	65.42 ± 11.90		
Urban and rural resident insurance	280	69.8	66.09 ± 11.57		
Commercial insurance	6	1.5	74.25 ± 5.63		
Lesion location				*H* = 0.546	0.909
Tongue	217	54.1	65.83 ± 11.42		
Buccal	128	31.9	66.19 ± 12.54		
Gingiva	41	10.2	66.55 ± 9.01		
Other	15	3.7	63.96 ± 13.90		
Pathological pattern				*U* = 3615.500	0.102
Squamous carcinoma	386	96.3	65.78 ± 11.62		
Other	15	3.7	70.32 ± 11.60		
Tumor stage				*H* = 84.522	< 0.001
I	30	7.5	71.02 ± 12.56		
II	139	34.7	71.51 ± 7.71		
III	128	31.9	65.01 ± 8.75		
IV	104	25.9	58.21 ± 14.02		
Chemoradio therapy				*U* = 12389.500	< 0.001
No	289	72.1	67.33 ± 10.92		
Yes	112	27.9	62.38 ± 12.69		
Complication				*U* = 13858.500	0.046
No	292	72.8	66.68 ± 11.36		
Yes	109	27.2	64.00 ± 12.19		

Abbreviations: *U*, Mann-Whitney U tests; *H*, Kruskal-Wallis H tests

### Regression analysis for covariate consideration

To examine which demographic or clinical characteristics were meaningfully associated with QoL, a multivariable linear regression including all 15 demographic and clinical variables was conducted (S1 Table 1). Three variables showed statistically significant associations: gender (β = −0.102, *P* = 0.025), caregiver identity (β = −0.103, *P* = 0.028), and tumor stage (β = −0.413, *P* < 0.001). Prior studies have shown that sociodemographic variables such as gender and caregiving roles may relate to QoL, but their effects are typically modest and inconsistent across samples [45]. In contrast, tumor stage is one of the most robust and consistently reported clinical determinants of postoperative QoL, strongly associated with symptom burden, functional decline, and recovery trajectories [46]. Given this well-established evidence and the substantially greater effect observed in our regression model, tumor stage was selected as the sole covariate for subsequent SEM.

### Correlation analysis

Table 2 presents the descriptive statistics and correlation analysis of the study variables. The mean score for QoL was 65.95 ± 11.64, while social alienation, body image, speech disorder, and self-esteem had mean scores of 32.23 ± 5.54, 4.81 ± 3.53, 33.59 ± 20.19, and 29.61 ± 3.41, respectively. Pearson correlation analysis revealed that QoL was negatively correlated with body image (*r* = − 0.566, *P* < 0.01), social alienation (*r* = − 0.557, *P* < 0.01), and speech disorder (*r* = − 0.681, *P* < 0.01), but positively associated with self-esteem (*r* = 0.535, *P* < 0.01), suggesting that poorer body image, higher social alienation, and greater speech impairments were linked to lower QoL, whereas higher self-esteem was associated with better QoL. Additionally, body image was positively correlated with social alienation (*r* = 0.717, *P* < 0.01) and speech disorder (*r* = 0.478, *P* < 0.01), while negatively correlated with self-esteem (*r* = − 0.642, *P* < 0.01). Social alienation showed a positive correlation with speech disorder (*r* = 0.521, *P* < 0.01) but was negatively associated with self-esteem (*r* = − 0.752, *P* < 0.01). Furthermore, speech disorder was inversely related to self-esteem (*r* = − 0.484, *P* < 0.01), indicating that greater speech difficulties were associated with lower self-esteem. These findings highlight the interconnections between body image, social alienation, speech function, and psychological well-being, emphasizing their collective impact on QoL.

**Table 2:** Correlation analysis of all study variables

Variables	M ± SD	1	2	3	4	5
1.Body image	4.81 ± 3.53	1				
2.Social alienation	32.23 ± 5.54	0.717**	1			
3.Speech disorder	33.59 ± 20.19	0.478**	0.521**	1		
4.Self-esteem	29.61 ± 3.41	−0.642**	−0.752**	-0.484**	1	
5.Quality of life	65.95 ± 11.64	−0.566**	−0.557**	−0.681**	0.535**	1

Abbreviations: **P < 0.01

### Exploratory factor analysis (EFA)

To ensure that the latent structures of the SES and UW-QOL were appropriate for this early postoperative oral cancer population, EFA was performed. This step was taken because confirmatory analyses of the original structures yielded suboptimal fit indices in this sample, suggesting potential divergence from the validation cohorts. The KMO measure for the SES was 0.856, and Bartlett’s test of sphericity produced a chi-square value of 1509.536, with a significance level of 0.000 < 0.001, indicating suitability for factor analysis. The first three components together accounted for 66.025% of the total variance after rotations (S1 Table 2). Based on the rotated component matrix (S1 Table 3), the SES was conceptualized as a latent variable encompassing three dimensions: sense of self-worth (items 1, 2, 3, 4, 5, 6, 7), self-denial (items 9, 10), and achievement motivation (item 8). For the UW-QOL, the KMO value was 0.851, and Bartlett’s test of sphericity yielded a chi-square value of 1313.684, with a significance level of 0.000 < 0.001, confirming the appropriateness of factor analysis. Following rotation, the first three components explained a cumulative variance of 54.615% (S1 Table 4). According to the rotated component matrix (S1 Table 5), the UW-QOL was structured as a latent variable comprising three dimensions: physical function (items 2, 3, 4, 11, 12), oral function (items 5, 6, 7), and symptoms and discomfort (items 1, 8, 9, 10)

### Structural equation modeling analysis

**Fig. 2:**
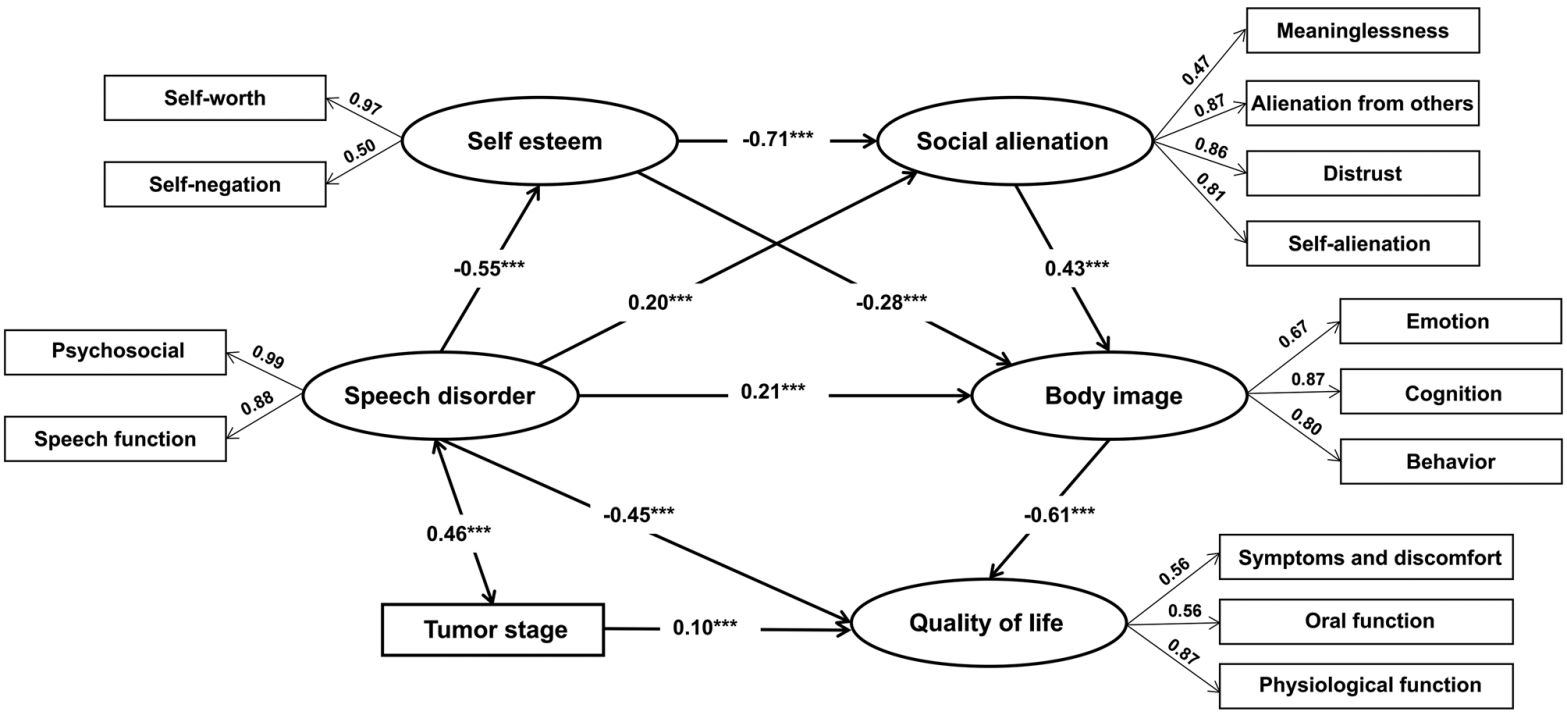
The final structural equation model evaluating the associations between body image, social alienation, speech disorder, self-esteem, and quality of life, with tumor stage included as a covariate. All path coefficients are standardized estimates. ***P < 0.001

The initial structural equation model for model fit testing is shown in S1 Fig. 1. The initial model, which did not include any covariates, demonstrated acceptable fit indices, as presented in 1 Table 6. However, the item “Achievement” showed a standardized factor loading of 0.31, which was below the commonly accepted threshold of 0.40 and was further indicated by the Wald test as contributing to poor model fit. Following this adjustment and incorporating tumor stage as the covariate identified in the regression analysis, the final model (Fig. 2) showed excellent fit: χ² = 187.818, df = 78, χ²/df = 2.408, GFI = 0.943, AGFI = 0.912, NFI = 0.949, CFI = 0.969, IFI = 0.970, TLI = 0.959, RMSEA = 0.059, and SRMR = 0.039. All indices met the established thresholds, indicating good model fit. The revised model maintained theoretical coherence while demonstrating improved parsimony and fit

In the final model, after adjusting for tumor stage, all hypothesized psychosocial path coefficients remained statistically significant (*P* < 0.001). Self-esteem showed no direct association with QoL, but was indirectly associated through two major pathways: via body image (β = 0.173, *P* < 0.001) and via social alienation and body image (β = 0.188, *P* < 0.001). Social alienation had no association with QoL, but showed an indirect negative association through body image (β = −0.264, *P* < 0.001). Speech disorder was strongly and negatively associated with QoL directly (β = −0.446, *P* < 0.001), along with additional indirect associations through four mediated routes: via body image (β = −0.126, *P* < 0.001), via social alienation and body image (β = −0.053, *P* < 0.001), via self-esteem and body image (β = −0.096, *P* < 0.001), and via self-esteem, social alienation, and body image (β = −0.104, *P* < 0.001). Body image exhibited a direct negative association with QoL (β = −0.609, *P* < 0.001), with no additional indirect pathways. These findings indicate that multiple layered mediation mechanisms exist among psychosocial variables associated with QoL, as detailed in Table 3.

**Table 3:** Standardized path model effect decomposition

Variables	Path	Direct effects	Indirect effects	Total effects
Self-esteem	SE → BI → QoL	—	0.173***	0.173***
	SE → SA → BI → QoL	—	0.188***	0.188***
Social alienation	SA → BI → QoL	—	−0.264***	−0.264***
Speech disorder	SD → QoL	−0.446***	−0.378***	−0.824***
	SD → BI → QoL	—	−0.126***	−0.126***
	SD → SA → BI → QoL	—	−0.053***	−0.053***
	SD → SE → BI → QoL	—	−0.096***	−0.096***
	SD → SE → SA → BI → QoL	—	−0.104***	−0.104***
Body image	BI → QoL	−0.609***	0.000***	−0.609***

Abbreviations: *SE*, Self-esteem; *SA*, Social alienation; *SD*, Speech disorder; *BI*, Body image; *QoL*, Quality of life. Standardized path coefficients are presented for the final model, adjusted for tumor stage. ***P < 0.001

## Discussion

Our study explored the factors associated with health-related QoL in patients following oral cancer surgery during the acute postoperative phase. By integrating speech function, body image, self-esteem, and social alienation into a single SEM framework, we provide evidence that QoL is not only related to functional impairments but also closely linked with psychosocial mechanisms such as internal evaluations and perceived connectedness. These results support a multidimensional understanding of survivorship, where functional, psychological, and social domains intersect in complex ways

In the initial analyses, several demographic and clinical characteristics were found to be significantly associated with postoperative QoL, including tumor stage, chemoradiotherapy status, marital status, residential type, caregiver identity, and presence of complications, consistent with prior evidence that advanced disease, intensive treatment, and limited social support increase vulnerability [47–49]. Recognizing these baseline disparities provides essential context for interpreting the complex psychosocial pathways subsequently identified in the structural equation model. A central insight of this research is the cascading mediation effect linking speech dysfunction to QoL through self-esteem, social alienation, and body image. Among all variables, body image exhibited the strongest direct association with QoL. Body image encompasses not only self-perception of appearance but also cognitive and emotional experiences related to bodily function [50, 51]. In oral cancer patients, both the disease and its surgical treatment frequently result in visible disfigurement and oral functional impairment [52], which in turn may lower self-esteem and increase feelings of social alienation. This sequence contributes to poorer body image [53], ultimately associated with lower QoL. Such a pathway is congruent with theoretical expectations and prior reports: head and neck cancer patients often experience clusters of problems, where speech and appearance changes undermine self-esteem, evoke stigma or rejection [54], and lead to negative body image and social withdrawal. These psychosocial repercussions have tangible consequences for patients’ daily functioning and social engagement. Notably, while speech dysfunction showed a direct negative association with QoL, the indirect associations through mediators were also substantial. This suggests that improving QoL may require attention not only to speech rehabilitation but also to psychological (e.g., self-esteem) and social (e.g., isolation) factors arising from speech impairment. In summary, the multiple mediation pathways identified here highlight how a single functional deficit can cascade through psychosocial layers to shape overall QoL

Our results are consistent with and extend previous research in head and neck oncology. Poorer body image showed the strongest direct association with reduced QoL in our model, aligning with numerous studies reporting that disfigurement-related distress is a key factor associated with well-being in cancer survivors [45]. The present study adds to this literature by situating these relationships in the acute postoperative phase, a period rarely examined in earlier work, when functional decline and psychological vulnerability may be especially pronounced. This is consistent with recent mixed-methods studies [53] suggesting that speech difficulties can contribute to body image concerns through psychological mediators. In addition, we employed EFA to redefine latent structures of the SES and UW-QOL, which enhanced model fit for this oral cancer sample. This analytic decision highlights that construct dimensionality may vary across populations and contexts, offering new insights into how psychosocial factors can be modeled. These results should therefore be interpreted as contributing context-specific evidence that complements, rather than replaces, prior findings based on original scale structures. It is also important to consider the predominantly male composition of our sample and the Chinese sociocultural context. In Chinese cancer populations, family caregiving expectations and cultural ideals of appearance have been shown to shape body image and psychosocial outcomes [55], suggesting that gender and culture may partly account for the observed associations

These findings carry important clinical implications, underscoring the need for early and integrated interventions in the postoperative management of oral cancer patients. The identification of a multi-step mediation pathway suggests that speech impairment is rarely an isolated problem, rather it is closely linked to reduced self-esteem, social disconnection, and body image dissatisfaction, which in turn impair QoL [16]. Clinically, this means that patients presenting with speech difficulties should be assessed not only for functional impairment but also for associated psychosocial distress, highlighting the need for comprehensive evaluation and intervention. Therefore, interventions should simultaneously address both functional and psychosocial dimensions. The acute postoperative phase, particularly the first week after surgery, represents a critical intervention window when patients are shaping perceptions of recovery and self-image [56]. Early rehabilitation of oral function is crucial [57], and studies have shown that prompt post-surgical interventions, such as swallowing and jaw mobility exercises, significantly improve outcomes and QoL [58]. Our finding extend this early intervention principles to the psychosocial realm: providing counseling or psychoeducation soon after surgery may help preserve self-esteem and prevent alienation [59, 60]. Additionally, family education, support groups, or peer mentorship programs can buffer against isolation [61]. This evidence supports a multidisciplinary model of care, where speech and swallowing therapy are combined with psychological counseling, social support, and dedicated body image interventions, given its strong association with QoL. Importantly, such strategies should be culturally adapted. In the Chinese sociocultural context, family caregiving expectations, work identity, and appearance norms may shape how self-esteem, social connectedness, and body image are experienced, and tailoring interventions to these cultural factors could enhance their acceptability and effectiveness. By targeting multiple links in the network of associations, including improving speech function, bolstering self-esteem, reducing social isolation, and addressing body image, clinicians may achieve a synergistic benefit on overall QoL. In summary, the study advocates for an early multidisciplinary intervention model in postoperative oral cancer care, one that treats the whole patient, not just the surgical site, thereby potentially improving long-term survivorship quality

Despite its strengths, this study has certain limitations. First, the cross-sectional design limits the ability to draw causal conclusions, and the reported pathways should be viewed as associations rather than definitive mechanisms. Second, the sample was predominantly male and drawn from a single center, which may restrict the extent to which the findings can be generalized to broader patient populations. Third, although validated instruments were used, some scales were modified through EFA, which may reduce strict comparability with earlier studies. In addition, one item of the SES with very low loading was removed during SEM optimization; while this improved model fit, it may also have affected the scale’s validity and comparability with prior work. Fourth, although tumor stage was included as a covariate in the SEM, other variables identified in the regression analysis were not incorporated due to sample imbalance and model parsimony considerations, so their potential influence on QoL cannot be completely ruled out. Fifth, reliance on self-report measures raises the possibility of recall bias and socially desirable responding. Finally, subgroup analyses were constrained by small group sizes, particularly among female and unmarried patients, which may have limited the ability to detect potential differences. Future research should employ longitudinal and multicenter designs, recruit more balanced samples, and further examine subgroup differences to strengthen the evidence base

## Conclusion

This study suggests that postoperative QoL in oral cancer patients is associated with an interplay of functional and psychosocial factors. The structural equation model revealed that speech dysfunction was negatively associated with QoL, both directly and indirectly through sequential pathways involving lower self-esteem, greater social alienation, and poorer body image. Among these variables, speech disorder showed the strongest overall association with QoL, while body image exerted the strongest direct effect. These findings underscore the importance of a multidimensional survivorship model and point to the potential value of early and integrated interventions that combine functional rehabilitation with psychosocial support. Future research should further explore these pathways using longitudinal and interventional designs to validate the associations observed and to inform clinical application

## Supplementary Information

Below is the link to the electronic supplementary material.

Supplementary Material 1

## Data Availability

The data that support the findings of this study are available on request from the corresponding author. The data are not publicly available due to privacy or ethical restrictions.
