# Supplementary material for: Structural equation modeling of multidimensional determinants of postoperative quality of life in patients with oral cancer
Source: Health Qual Life Outcomes. 2026 Jan 22;24:20. doi: 10.1186/s12955-026-02476-1 (PMC12908360; doi:10.1186/s12955-026-02476-1)
Supplement: Supplementary file 1 — Supplementary Material 1 [file 12955_2026_2476_MOESM1_ESM.docx]

**S1 Table 1**. Multivariable Linear Regression Analysis of Factors Associated with Quality of Life (N = 401).

| **Variables** | **B** | **SE** | **Beta** | **t** | ***P*** | **VIF** |
| --- | --- | --- | --- | --- | --- | --- |
| Constant | 76.268 | 5.747 |  | 13.271 | <.001 |  |
| Age | 0.085 | 0.721 | 0.007 | 0.119 | 0.906 | 1.524 |
| Gender | -4.112 | 1.826 | -0.102 | -2.252 | 0.025 | 1.043 |
| Marital status | 0.179 | 3.927 | 0.003 | 0.046 | 0.964 | 2.858 |
| Habitation | 1.263 | 1.254 | 0.054 | 1.007 | 0.314 | 1.462 |
| Residential method | -2.936 | 3.648 | -0.06 | -0.805 | 0.421 | 2.798 |
| Personal monthly income (RMB) | 1.015 | 0.609 | 0.093 | 1.666 | 0.096 | 1.557 |
| Educational level | 0.171 | 0.707 | 0.013 | 0.242 | 0.809 | 1.492 |
| Occupation | 0.259 | 1.018 | 0.014 | 0.254 | 0.799 | 1.427 |
| Caregiver identity | -1.679 | 0.759 | -0.103 | -2.211 | 0.028 | 1.087 |
| Type of health insurance | 0.465 | 0.979 | 0.023 | 0.475 | 0.635 | 1.182 |
| Lesion location | -0.06 | 0.663 | -0.004 | -0.091 | 0.928 | 1.084 |
| Pathological pattern | 3.391 | 2.852 | 0.055 | 1.189 | 0.235 | 1.094 |
| Tumor stage | -5.211 | 0.623 | -0.413 | -8.361 | <.001 | 1.232 |
| Chemoradio therapy | -0.606 | 1.272 | -0.023 | -0.476 | 0.634 | 1.217 |
| Complication | -1.862 | 1.209 | -0.071 | -1.54 | 0.124 | 1.082 |

Abbreviations: *B*, unstandardized regression coefficient. *SE*, standard error. *Beta*, standardized regression coefficient. *VIF*, variance inflation factor. Bolded *P* < 0.05 indicates statistical significance. Dependent variable: QOL.

**S1 Table 2**. Variance Explanation Results of Principal Component Analysis for the Self-Esteem Scale.

| **Total Variance Explained** | | | | | | | | | |
| --- | --- | --- | --- | --- | --- | --- | --- | --- | --- |
| Component | Initial Eigenvalues | | | Extraction Sums of Squared Loadings | | | Rotation Sums of Squared Loadings | | |
|  | Total | % of Variance | Cumulative % | Total | % of Variance | Cumulative % | Total | % of Variance | Cumulative % |
| 1 | 4.426 | 44.255 | 44.255 | 4.426 | 44.255 | 44.255 | 3.318 | 33.184 | 33.184 |
| 2 | 1.191 | 11.908 | 56.163 | 1.191 | 11.908 | 56.163 | 1.877 | 18.769 | 51.953 |
| 3 | 0.986 | 9.862 | 66.025 | 0.986 | 9.862 | 66.025 | 1.407 | 14.072 | 66.025 |
| 4 | 0.708 | 7.08 | 73.105 |  |  |  |  |  |  |
| 5 | 0.638 | 6.379 | 79.484 |  |  |  |  |  |  |
| 6 | 0.579 | 5.788 | 85.272 |  |  |  |  |  |  |
| 7 | 0.465 | 4.645 | 89.918 |  |  |  |  |  |  |
| 8 | 0.443 | 4.428 | 94.346 |  |  |  |  |  |  |
| 9 | 0.314 | 3.137 | 97.482 |  |  |  |  |  |  |
| 10 | 0.252 | 2.518 | 100 |  |  |  |  |  |  |

**S1 Table 3.** Rotated Component Matrix of the Self-Esteem Scale.

| **Component Matrixa** | | | |
| --- | --- | --- | --- |
|  | Component | | |
|  | 1 | 2 | 3 |
| 5.I do not have many reasons to feel proud of myself. | 0.773 |  | -0.154 |
| 3.At the end of the day, I tend to see myself as a failure. | 0.747 | 0.219 |  |
| 6.At the end of the day, I tend to see myself as a failure. | 0.68 | 0.125 | 0.411 |
| 1.I feel that I am a person of worth, at least on an equal level with others. | 0.654 | 0.289 | 0.112 |
| 7.I have a positive attitude toward myself. | 0.641 | 0.172 | 0.511 |
| 2.I feel that I have many good qualities. | 0.63 | 0.21 | 0.244 |
| 4.I am capable of doing things as well as most people. | 0.599 | 0.233 | 0.194 |
| 9.I often feel that I am useless. | 0.183 | 0.91 |  |
| 10.I frequently think that I am worthless. | 0.259 | 0.877 | 0.134 |
| 8.I wish I could earn more respect for myself. |  |  | 0.906 |

**S1 Table 4**. Variance Explanation Results of Principal Component Analysis for the Quality of Life Scale.

| **Total Variance Explained** | | | | | | | | | |
| --- | --- | --- | --- | --- | --- | --- | --- | --- | --- |
| Component | Initial Eigenvalues | | | Extraction Sums of Squared Loadings | | | Rotation Sums of Squared Loadings | | |
|  | Total | % of Variance | Cumulative % | Total | % of Variance | Cumulative % | Total | % of Variance | Cumulative % |
| 1 | 4.161 | 34.674 | 34.674 | 4.161 | 34.674 | 34.674 | 2.949 | 24.576 | 24.576 |
| 2 | 1.386 | 11.552 | 46.226 | 1.386 | 11.552 | 46.226 | 1.994 | 16.62 | 41.196 |
| 3 | 1.007 | 8.389 | 54.615 | 1.007 | 8.389 | 54.615 | 1.61 | 13.419 | 54.615 |
| 4 | 0.917 | 7.641 | 62.256 |  |  |  |  |  |  |
| 5 | 0.836 | 6.969 | 69.225 |  |  |  |  |  |  |

**S1 Table 5.** Rotated Component Matrix of the Quality of Life Scale.

| **Component Matrixa** | | | |
| --- | --- | --- | --- |
|  | Component | | |
|  | 1 | 2 | 3 |
| 12.Anxiety | 0.804 | 0.183 |  |
| 2.Appearance | 0.802 |  |  |
| 11.Emotion | 0.745 | 0.341 | 0.121 |
| 3.Activity | 0.634 | 0.289 | 0.242 |
| 4.Recreation | 0.491 | 0.265 | 0.369 |
| 5.Swallowing |  | 0.817 |  |
| 6.Chewing | 0.181 | 0.732 | 0.105 |
| 7.Speech | 0.369 | 0.659 |  |
| 1.Pain |  | 0.14 | 0.685 |
| 10.Saliva |  |  | 0.648 |
| 9.Taste | 0.385 | 0.15 | 0.523 |
| 8.Shoulder | 0.37 |  | 0.467 |


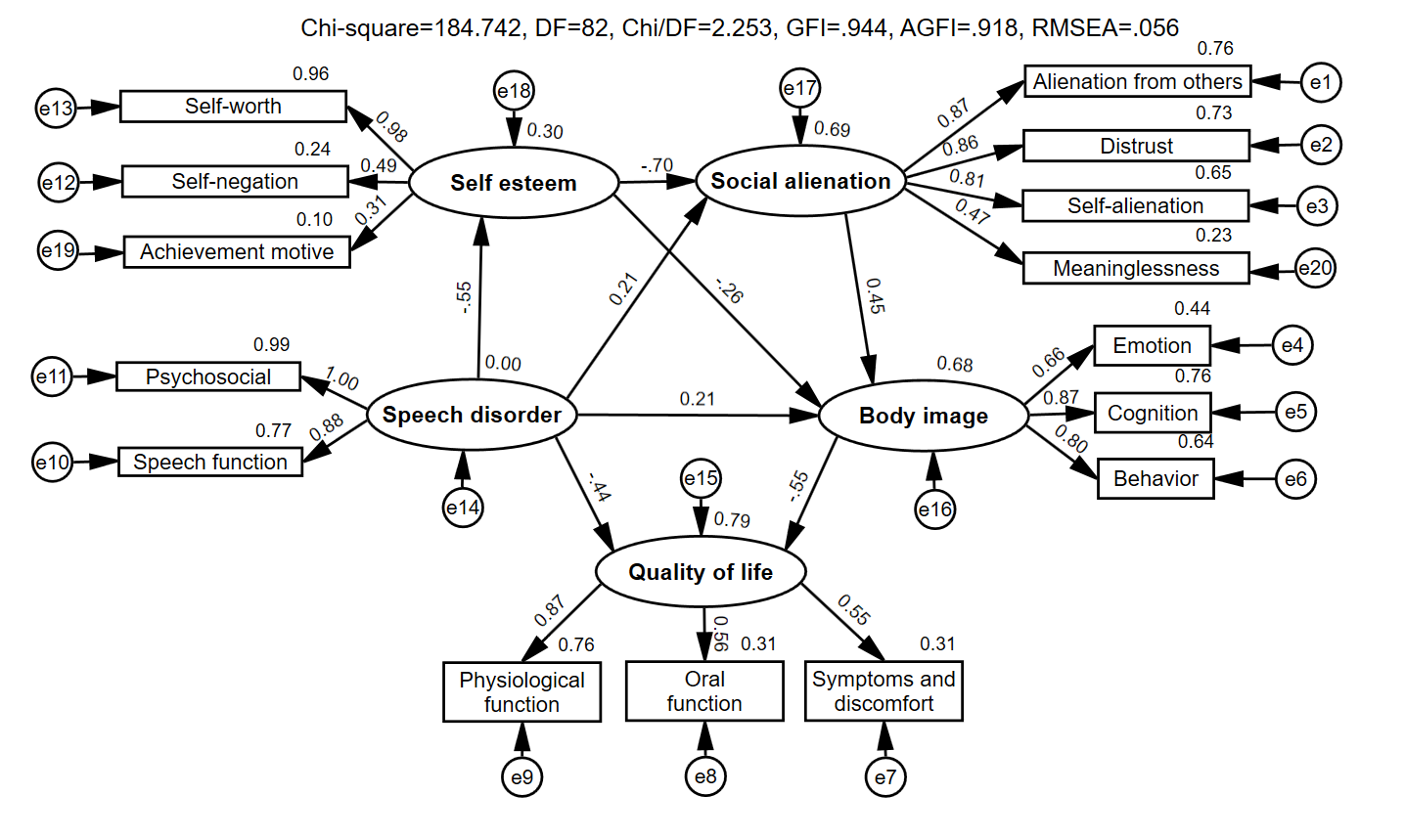


**S1 Figure 1**. The initial structural equation model evaluating the associations between Body image, Social alienation, Speech disorder, Self-esteem, and Quality of life. All path coefficients are standardized estimates.

**S1 Table 6.** The model fit indices of the initial model

| **Fit Indices** | **NC-χ2/DF（DF=82）** | **GFI** | **AGFI** | **NFI** | **CFI** | **IFI** | **TLI** | **RMSEA** | **SRMR** |
| --- | --- | --- | --- | --- | --- | --- | --- | --- | --- |
| Fit Criteria | ＜3 | ＞0.9 | ＞0.9 | ＞0.9 | ＞0.9 | ＞0.9 | ＞0.9 | ＜0.08 | ＜0.08 |
| Modified Model | 2.253 | 0.944 | 0.918 | 0.947 | 0.970 | 0.970 | 0.961 | 0.056 | 0.0394 |

**S1 Table 7**. Path analysis of quality of life determinants in postoperative oral cancer patients.

| **Model path** | | | **Unstd. Est** | **Est** | **S.E.** | **C.R.** | **P** |
| --- | --- | --- | --- | --- | --- | --- | --- |
| Self-esteem | <--- | Speech disorder | -0.161 | -0.553 | 0.013 | -11.897 | *** |
| Social alienation | <--- | Self-esteem | -0.483 | -0.713 | 0.056 | -8.633 | *** |
| Social alienation | <--- | Speech disorder | 0.039 | 0.199 | 0.011 | 3.583 | *** |
| Body image | <--- | Speech disorder | 0.022 | 0.207 | 0.005 | 4.283 | *** |
| Body image | <--- | Social alienation | 0.231 | 0.433 | 0.052 | 4.447 | *** |
| Body image | <--- | Self-esteem | -0.102 | -0.284 | 0.035 | -2.915 | .004 |
| Quality of life | <--- | Speech disorder | -0.269 | -0.446 | 0.041 | -6.482 | *** |
| Quality of life | <--- | Tumor stage | 0.318 | 0.101 | 0.318 | 1.953 | .051 |
| Quality of life | <--- | Body image | -3.504 | -0.609 | 0.053 | -6.970 | *** |
| Alienation from others | <--- | Social alienation | 1.000 | 0.872 | — | — | — |
| Distrust | <--- | Social alienation | 0.914 | 0.862 | 0.042 | 21.875 | *** |
| Self-alienation | <--- | Social alienation | 0.657 | 0.805 | 0.033 | 20.159 | *** |
| Emotion | <--- | Body image | 1.000 | 0.659 | — | — | — |
| Cognition | <--- | Body image | 1.489 | 0.875 | 0.102 | 14.564 | *** |
| Behavior | <--- | Body image | 0.737 | 0.797 | 0.052 | 14.061 | *** |
| Speech function | <--- | Speech disorder | 1.000 | 0.880 | — | — | — |
| Psychosocial | <--- | Speech disorder | 1.073 | 0.895 | 0.040 | 26.640 | *** |
| Symptoms and discomfort | <--- | Quality of life | 1.000 | 0.552 | — | — | *** |
| Oral function | <--- | Quality of life | 1.858 | 0.560 | 0.214 | 8.677 | *** |
| Physiological function | <--- | Quality of life | 2.391 | 0.872 | 0.220 | 10.858 | *** |
| Meaninglessness | <--- | Social alienation | 0.243 | 0.473 | 0.025 | 9.764 | *** |
| Self-worth | <--- | Self-esteem | 1.000 | 0.961 | — | — | — |
| Self-negation | <--- | Self-esteem | 0.139 | 0.945 | 0.015 | 9.16 | *** |

Abbreviations: *Unstd. Est*, Unstandardized Estimate; *Est*, Estimate. ***p < 0.001.
